# DiabCompSepsAI: Integrated AI Model for Early Detection and Prediction of Postoperative Complications in Diabetic Patients—Using a Random Forest Classifier

**DOI:** 10.3390/jcm14207173

**Published:** 2025-10-11

**Authors:** Sri Harsha Boppana, Sachin Sravan Kumar Komati, Raja Hamsa Chitturi, Ritwik Raj, C. David Mintz

**Affiliations:** 1 Department of Internal Medicine, Nassau University Medical Center, East Meadow, NY 11554, USA; sboppan1@numc.edu; 2 Department of Computer Science, Florida International University, Miami, FL 33199, USA; sachinkomati23@gmail.com; 3 Department of Internal Medicine, HCA Florida Ocala Hospital, Ocala, FL 34471, USA; rajahamsa.chitturi@hcahealthcare.com; 4 Zanvyl Krieger School of Arts and Sciences, Johns Hopkins University, Baltimore, MD 21218, USA; rraj4@jh.edu; 5 Department of Anesthesiology & Critical Care Medicine, Johns Hopkins School of Medicine, Baltimore, MD 21205, USA

**Keywords:** postoperative complications, diabetic patients, wound infections, sepsis, random forest classifier, clinical risk assessment

## Abstract

**Background/Objectives:** Postoperative complications such as wound infections and sepsis are common in diabetic patients, often resulting in longer hospital stays and higher morbidity. This study hypothesizes that a Random Forest Classifier can accurately predict these complications, enabling early clinical interventions. The model utilizes ensemble learning to integrate diverse patient data and improve predictive accuracy beyond traditional risk assessments. **Methods:** A comprehensive retrospective analysis was performed using data extracted from the National Surgical Quality Improvement Program (NSQIP) database. The dataset encompassed a wide array of variables, including demographic factors, clinical markers, and detailed surgical data (specialty, type of anesthesia, duration of surgery). Each variable was meticulously encoded into numerical formats, with categorical variables transformed through one-hot encoding, and continuous variables were normalized. The dataset was partitioned into training (80%) and testing (20%) subsets, ensuring a balanced representation of the target outcomes. The Random Forest Classifier was selected due to its robustness in handling high-dimensional data and its ability to model complex interactions between variables. **Results:** The Random Forest model showed accuracy rates of 94.38% for wound infection and 94.94% for sepsis. Precision and recall metrics also exceeded 94%, highlighting the model’s accuracy in identifying true positives and reducing false positives. ROC curve analysis yielded AUC values of 0.92 for wound infection and 0.95 for sepsis, indicating strong discriminative capability. Feature importance analysis further identified key predictors, including surgical duration, specific laboratory markers, and patient comorbidities. **Conclusions:** This study demonstrates the Random Forest Classifier’s strong predictive ability for postoperative wound infections and sepsis in diabetic patients. The model’s high-performance metrics indicate its potential for real-time risk stratification in clinical workflows. Future research should validate these findings in diverse populations and surgical settings. Incorporating this predictive model into clinical practice has the potential to significantly improve patient outcomes and reduce healthcare costs.

## 1. Introduction

Globally, an estimated five billion people, about two-thirds of the world’s population, still lack access to safe, affordable, and timely surgical and anesthesia care, underscoring the ongoing scale of unmet surgical need [1,2]. This access gap and the rising burden of surgically treatable conditions in low- and middle-income countries continue to impose substantial health and economic costs; recent analyses and reviews highlight persistent delays, workforce shortages, and large volumes of unmet procedures [1,2]. These postoperative complications not only impact individual patients but also impose significant socioeconomic burdens, increasing treatment costs by 119–172% compared to cases with uncomplicated recoveries [3]. In the context of rising healthcare costs and constrained financial resources, identifying patients at risk of postoperative complications and implementing personalized, precision medicine-based treatment strategies both offer a promising approach to reducing patient morbidity, mortality, and healthcare-related expenses.

Postoperative complications, such as wound infections and sepsis, pose significant risks to patients undergoing surgery, particularly those with diabetes mellitus. Diabetes is a well-established risk factor for adverse surgical outcomes due to its association with impaired wound healing and immune response [4]. The accurate prediction of these complications is crucial for timely interventions and improved patient management. SSIs continue to pose a significant challenge, contributing substantially to postoperative morbidity and mortality. It is estimated that over 2 million nosocomial infections in the United States are due to SSIs each year, underscoring the burden on healthcare systems [5].

Sepsis continues to be a major concern in the postoperative period, significantly affecting patient recovery and healthcare resources. This serious condition, which results from an infection and can lead to organ failure, often develops after surgical site infections (SSIs) but can stem from various other infections. In the U.S., sepsis is linked to a high number of hospitalizations and fatalities, contributing to approximately 270,000 deaths annually [6]. Early identification and swift treatment are crucial to prevent sepsis from worsening, limit organ damage, and reduce the high mortality and healthcare costs associated with the condition.

Diabetes mellitus (DM) is among the most impactful chronic diseases globally, particularly in developing and newly industrialized countries [7]. Among the various types of diabetes, Type 2 DM (DM 2) is the most prevalent, accounting for 90% of all cases, and is notably associated with insulin resistance [8]. It has been noted for some time that patients with diabetes are at an increased risk for postoperative complications, particularly surgical site infections (SSIs) and sepsis. Historically, before the acceptance of germ theory and the adoption of antiseptic techniques, the incidence of postoperative infections was alarmingly high, often leading to severe outcomes such as limb amputation or death [9,10]. While the risks of DM with elevated rates of SSIs are well-known at the population level, there are few prognostic resources for clinicians seeking to predict whether their patient is likely to suffer from this potentially devastating complication in the hopes of obtaining a better outcome.

This study aims to develop a predictive model using Random Forest to assess the risk of SSI and sepsis in patients with DM following surgery. By leveraging a comprehensive dataset from NSQIP and employing rigorous data preprocessing and feature selection methods, this research seeks to provide a robust tool for clinicians to identify high-risk patients and implement preventive measures.

## 2. Methods

### 2.1. Data Collection

This study draws upon data collected from the NSQIP, predominantly U.S.-based, over the period from 2014 to 2020, offering an extensive dataset that includes essential patient demographics and clinical variables. Since this study utilized data from the NSQIP, ethical approval was not necessary. NSQIP is a de-identified, available to qualified investigators from ACS upon application under standard terms, created especially for surgical outcome research and quality improvement. Data were obtained from the NSQIP Participant Use File through a participating institution’s standard access pathway. The use of NSQIP data complies with privacy regulations and ethical guidelines for research involving human participants’ data, eliminating the need for additional ethics review. The NSQIP dataset was accessed on 23 August 2023.

The data used in this study comprises 17,000 records of diabetic patients. Each record includes 22 features aimed at predicting postoperative complications, providing a broad dataset for analysis. The study sample consists of adult patients aged between 18 and 90 years, with a mean age of approximately 59 years. These individuals underwent various surgical procedures across 10 medical specialties. The dataset includes demographic details, surgical information, and preoperative laboratory values. Both elective and emergency surgeries were included in the dataset, with 84.8% classified as elective and 15.2% as emergency procedures. Notably, the dataset contained no missing values, allowing for comprehensive analysis without the need for exclusions. This inclusive approach supports a thorough investigation of postoperative complications, such as wound infections and sepsis, in a high-risk diabetic population undergoing diverse surgical interventions.

### 2.2. Features

The 22 features selected for model training cover a wide range of clinical, demographic, and procedural details, including: Demographics (sex and age), Clinical Markers (admission type [inpatient or outpatient], transfer status, type of anesthesia, discharge destination, height, and weight), Surgical Details (surgical specialty, elective or emergency surgery status, surgery duration [operation time], and wound classification), Patient History (smoking status, presence of dyspnea [difficulty breathing], cancer diagnosis, and diabetes status), Laboratory values (preoperative serum sodium [mmol/L; dprna], serum albumin [g/dL; dpralbum], and hematocrit [%; dprhct], all obtained within 24–48 h before the index procedure), and other clinical factors (emergency status of the surgery, deep renal insufficiency, and steroid use).

### 2.3. Feature Selection

Features included in the model were chosen based on their clinical relevance and potential impact on predicting postoperative complications. Key categories considered were demographics, clinical markers, surgical details, patient history, laboratory values, and other clinical factors [5,6,7,8,9]. This methodical selection process focused on variables with the highest clinical significance, enhancing the model’s precision and reliability in predicting outcomes such as wound infection and sepsis.

The primary outcome measures in this study were the occurrence of wound infection and sepsis. These complications were selected as target variables due to their clinical relevance in evaluating postoperative recovery and overall patient health after surgery.

### 2.4. Datasets

This study’s primary objective was to develop and validate a predictive model for postoperative complications, specifically targeting wound infection and sepsis in patients undergoing surgery. By identifying factors linked to these complications, the model aims to support early interventions and improve patient outcomes. For model development, the data was divided into training and testing sets: The training set accounted for 80% of the total records (13,600 patients) while the testing set comprised 20% of the total records (3400 patients).

**Figure 1:**
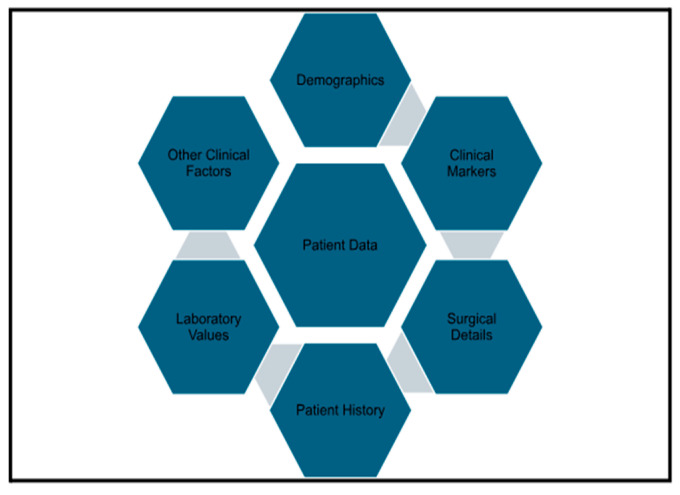
Hexagonal Visualization of Patient Data Categories.

**Figure 2:**
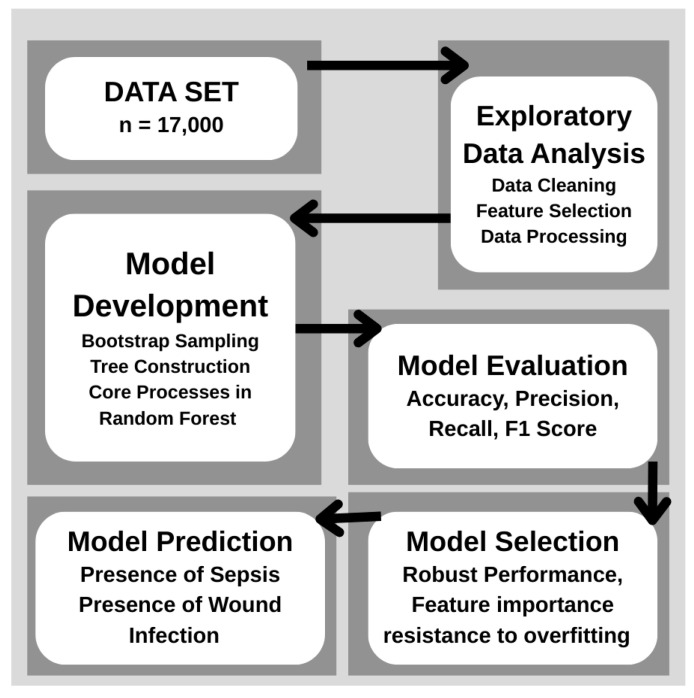
Workflow of the model.

This data split provided a robust basis for evaluating the model’s accuracy on new, unseen data. The trained model’s performance on the test set offered insights into its predictive reliability and capacity for generalization across different patient cases. The primary data categories included in the model to predict postoperative complications in diabetic patients. The sequence of steps from data collection to model training and deployment. Key stages include data preprocessing, feature selection, validation, and integration into the Streamlit application for real-time prediction. Figure 1 provides a hexagonal visualization of patient data categories. Figure 2 depicts the workflow of the model.

## 3. Data Preprocessing

To prepare the dataset for analysis, categorical variables were converted into numerical representations, allowing the machine learning model to process and learn from these variables effectively. This encoding approach was essential to retain the inherent distinctions and relationships within each category, which aids the model in generating reliable predictions.

Binary variables, specifically those indicating the presence or absence of conditions such as diabetes status and smoking status, were encoded with straightforward numerical labels: a value of 0 for “No” and 1 for “Yes.” This binary encoding allowed the model to differentiate between these two states efficiently.

Sex was another categorical variable that required encoding. Here, male and female were represented by 0 and 1, respectively. This binary classification facilitated the model’s recognition of gender-based differences in outcomes without introducing additional complexity.

The status of patients as either inpatient or outpatient was also encoded numerically, with inpatients assigned a value of 0 and outpatients a value of 1. Similarly, transfer status, which indicated whether a patient was admitted from home or transferred from another facility, was encoded to capture various sources of admission. Specifically, values ranged from 0 for patients admitted from home to 1 for those transferred from an acute care hospital, 2 for those from outside emergency departments, and 3 for other sources. This categorization provided the model with a nuanced understanding of admission origins and their potential impact on outcomes.

Discharge destination was another key categorical variable, capturing where patients were discharged post-procedure. Values were assigned to distinguish between common destinations: 0 for home, 1 for other destinations, 2 for rehabilitation centers, 3 for skilled care settings outside the home, and 4 for unknown destinations. By distinguishing discharge outcomes, the model could assess how these destinations might influence patient recovery.

The type of anesthesia administered was similarly encoded to reflect different anesthesia types used in the procedures. The numerical encoding ranged from 0 for general anesthesia to 1 for monitored anesthesia care or intravenous sedation, 2 for spinal anesthesia, and 3 for other types. Encoding this variable allowed the model to account for the potential effects of anesthesia type on postoperative outcomes.

Surgical specialty, indicating the department under which a procedure was conducted, was also encoded numerically. Each specialty, such as urology, orthopedics, or general surgery, was assigned a distinct value, enabling the model to consider specialty-specific factors. For instance, urology was encoded as 0, orthopedics as 1, general surgery as 2, neurosurgery as 3, thoracic surgery as 4, otolaryngology (ENT) as 5, vascular surgery as 6, plastic surgery as 7, cardiac surgery as 8, and gynecology as 9. This distinction helped the model process data across various specialties with improved precision.

For diabetes medication, which indicated whether a patient was on insulin, non-insulin medications, or neither, encoding values ranged from 0 for no medication, 1 for non-insulin medications, and 2 for insulin. This provided the model with an understanding of the type of diabetes management in place, which could be a predictor of postoperative complications.

Dyspnea severity was another variable encoded based on patient symptoms, with values indicating no dyspnea, dyspnea on moderate exertion, or dyspnea at rest. Specifically, 0 was used for no dyspnea, 1 for moderate exertion, and 2 for dyspnea at rest. This classification enabled the model to assess the severity of respiratory symptoms, which could influence surgical outcomes.

Finally, wound classification, which categorized the wound’s degree of contamination, was encoded to reflect levels of cleanliness. This variable used values from 0 for clean or clean/contaminated wounds, 1 for clean, 2 for dirty or infected, and 3 for contaminated. This categorization helped the model recognize the risk factors associated with each level of wound contamination.

By encoding these categorical variables into distinct numerical values, the machine learning model could leverage them as predictors in the analysis. This structured approach enhanced the model’s ability to predict postoperative complications, such as wound infection and sepsis, with increased accuracy.

## 4. Model Development

### 4.1. Random Forest Classifier

In this study, we employed a random forest classifier to create a robust predictive model. The random forest algorithm, an ensemble learning method for classification, regression, and various other tasks, operates by constructing multiple decision trees during training. This approach allows for enhanced predictive accuracy by combining the outputs of numerous trees, each constructed from different data subsets.

### 4.2. Random Forest Functionality

The random forest classifier is a widely used supervised machine learning algorithm for classification and regression tasks. It builds multiple decision trees from different data samples, which collectively improve the model’s accuracy. For classification tasks, the final prediction is made through a majority vote across trees, while for regression tasks, an average of the trees’ outputs is taken. This ensemble technique reduces the likelihood of overfitting and improves the model’s generalization.

### 4.3. Mechanics of the Random Forest Algorithm

**Figure 3:**
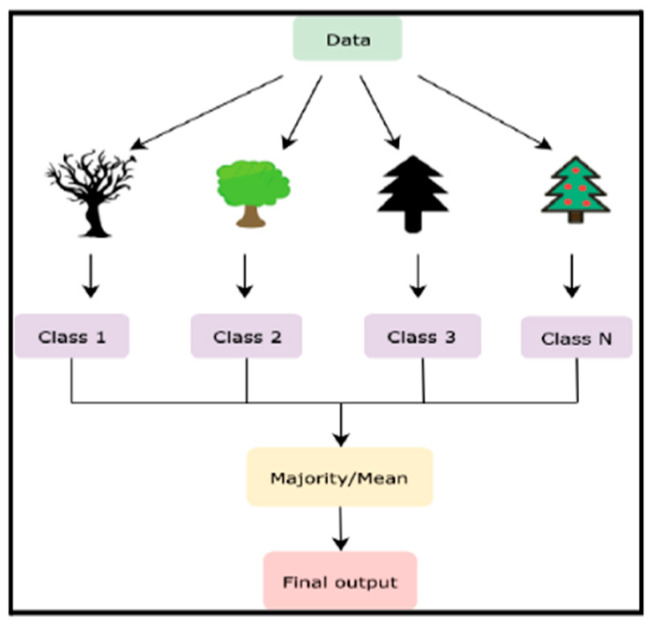
Decision Tree in Random Forest: Example decision tree within the Random Forest model. This figure highlights the stepwise process of splitting features to make predictions.

The random forest algorithm begins with constructing multiple decision trees, each trained on a distinct subset of data obtained via bootstrap sampling. During training, a random subset of features is chosen at each decision node, ensuring diversity among the trees. The final prediction is an aggregate result from all trees, either by taking the majority vote for classification or calculating the mean for regression. This process, illustrated in Figure 3, highlights the steps involved in developing a random forest model, from data sampling to final prediction aggregation.

### 4.4. Algorithm Selection

After comparative analysis with other algorithms, including logistic regression, support vector machines, and neural networks, we selected the random forest classifier. This choice was driven by its capability to handle complex data interactions, provide feature-importance insights, and resist overfitting. The random forest model outperformed others in terms of accuracy, precision, and recall for predicting postoperative complications, such as wound infection and sepsis, making it particularly suitable for our objectives.

### 4.5. Core Processes in Random Forest Model Development

The development of the random forest model involves several key steps. First, bootstrap sampling was used to create data subsets. For each tree in the forest, a unique training subset is created by sampling the data with replacement. This process ensures that each tree is exposed to slightly different data patterns, fostering diversity within the model.

The second step involved feature selection. At each node within a tree, a random subset of features is selected, and the optimal split is chosen from this subset. This randomization further reduces correlations between the trees, promoting greater model generalizability.

**Figure 4:**
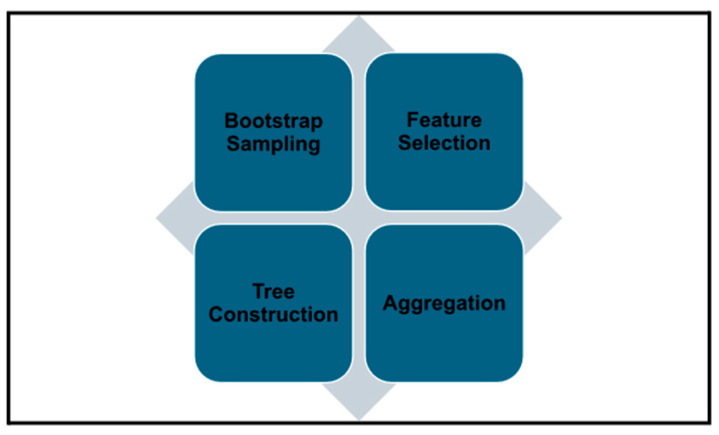
Core Processes in Random Forest Model Development: Core processes in Random Forest model development.

Finally, each tree is grown to its maximum depth without pruning, preserving its individual predictive potential. The variability introduced through bootstrap sampling and feature selection contributes to the trees’ uncorrelated nature. Figure 3 shows an example decision tree within the Random Forest model, highlighting the stepwise process of splitting features to generate predictions. Figure 4 illustrates the core processes involved in developing the Random Forest model.

## 5. Results

### 5.1. Model Performance

The predictive model’s effectiveness was evaluated using several key classification metrics, including accuracy, precision, recall, F1-score, and area under the receiver operating characteristic curve (ROC-AUC). These metrics together provide a comprehensive overview of the model’s classification performance in identifying cases of wound infection (wndinf) and sepsis (prsepis).

Accuracy: The model demonstrated strong classification performance, achieving an accuracy of 94% for wound infection cases and 94.8% for sepsis cases. These high-accuracy values indicate the model’s reliability in correctly classifying both conditions.

Precision: The precision scores were 93.6% for wound infection and 94.3% for sepsis, suggesting a high degree of accuracy in positive predictions for both target conditions. Precision reflects the proportion of positive identifications that are correct, emphasizing the model’s capability to limit false positive results.

Recall: With recall scores of 94% for wound infection and 94.8% for sepsis, the model effectively identified true positive cases. Recall measures the proportion of actual positives accurately detected by the model, underlining its sensitivity in recognizing cases of both wound infection and sepsis.

**Figure 5:**
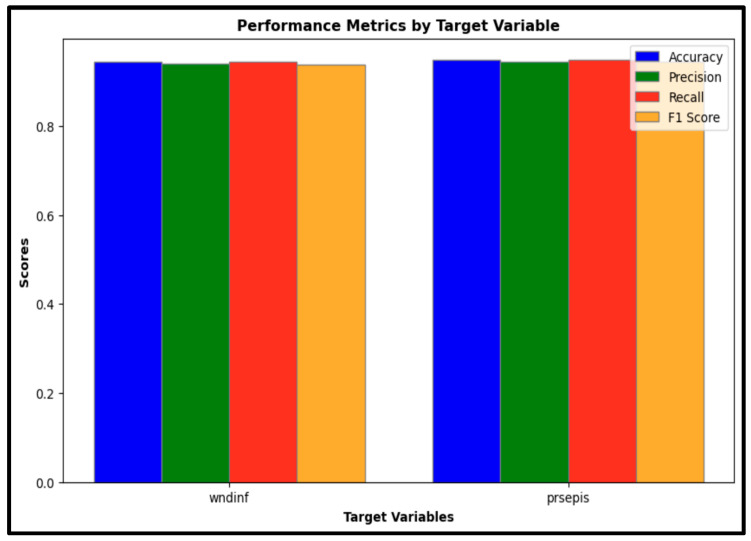
Performance Metrics by Target Variable: A comparative analysis of accuracy, precision, recall, and F1-scores for predicting wound infection (wndinf) and sepsis (prsepis). Metrics demonstrate strong classification performance across both conditions.

F1-Score: The F1-scores were 93.4% for wound infection and 94.3% for sepsis. As a harmonic mean of precision and recall, the F1-score offers a balanced metric to evaluate the model’s overall predictive performance, combining both sensitivity and specificity in one measure. Figure 5 presents a comparative analysis of accuracy, precision, recall, and F1-scores for wound infection (wndinf) and sepsis (prsepis), demonstrating that the model maintains strong classification performance across both outcomes.

The performance metrics are compiled in Table 1, offering a detailed breakdown of the model’s capabilities in classifying wound infection and sepsis cases across these key metrics.

### 5.2. Validation Results

The model was validated using a separate test dataset, comprising 3400 records. The results confirmed the model’s robustness, with high accuracy and consistency across various metrics. Confidence intervals and statistical significance (*p*-values) were calculated to ensure the reliability of the findings.

### 5.3. Receiver Operating Characteristic (ROC) Curve

**Figure 6:**
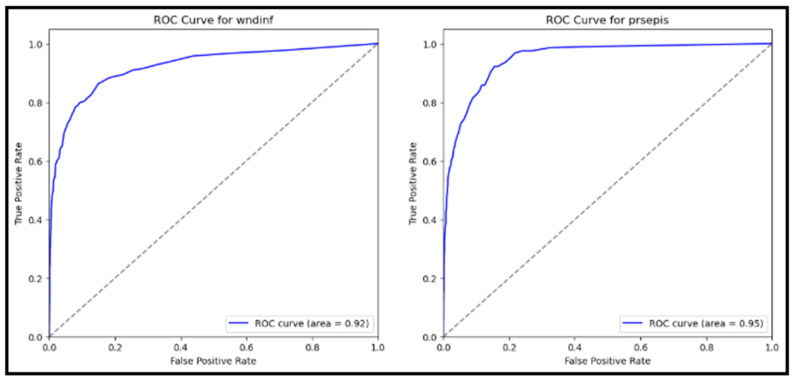
ROC-AUCs for Wound Infection and Sepsis Prediction: Receiver operating characteristic (ROC) curves for wound infection and sepsis prediction. The curves demonstrate the model’s discriminative ability, with ROC-AUC scores of 0.92 for wound infection and 0.95 for sepsis, indicating high sensitivity.

The ROC curves for both wound infection and sepsis are depicted in Figure 6. The Area Under the ROC Curve (AUC) scores were 0.92 for wound infection and 0.95 for sepsis. These high AUC values indicate excellent discriminative ability, with both curves approaching the upper left corner of the plot. The ROC-AUC for Wound Infection (wndinf) was 0.92, and the ROC-AUC for Sepsis (prsepis) was 0.95.

These results demonstrate the model’s strong performance in distinguishing between patients with and without postoperative complications. The model evaluation results demonstrate robust performance in predicting postoperative complications in diabetic patients. High scores across all metrics for wound infection (wndinf) and sepsis (prsepis) were achieved, with accuracy, precision, recall, and F1-scores all approaching 94%. The high precision scores indicate the model’s ability to minimize false positives, ensuring that positive predictions are highly accurate. The ROC curves, with AUC values of 0.92 for wound infection and 0.95 for sepsis, further validate the model’s strong discriminative ability. These results highlight the model’s potential utility in clinical settings for early risk stratification and targeted interventions, ultimately enhancing patient care and outcomes (Figure 6).

## 6. Demographic and Feature Influence

### 6.1. Feature Importance Analysis

The analysis of feature importance offers valuable insights into the variables most significantly influencing model predictions for postoperative complications. Below is a detailed description of the primary factors identified:

1. Operation Time (optime): Among the analyzed features, operation time proved to be the most influential, suggesting that extended surgical durations may correlate with an increased risk of postoperative complications. This finding implies that reducing operation time, where feasible, may be crucial in minimizing postoperative risks.
2. Weight: Patient weight emerged as a critical factor, likely due to its association with multiple health conditions that can impact recovery and elevate complication rates. This association underscores the importance of weight management and risk assessment in surgical planning.
3. Wound Classification (wndclas): The classification of wounds (e.g., clean, contaminated) significantly affects the likelihood of complications, as certain types are more susceptible to infection. This factor highlights the importance of wound management and postoperative care based on wound classification.
4. Age: Age was another prominent factor, with older patients showing a heightened risk of complications. This trend likely reflects the increased prevalence of comorbidities and slower recovery rates in older populations, necessitating more comprehensive risk management strategies for elderly patients.
5. Surgical Specialty (surgspec): The specialty of the surgery also contributed to variation in complication rates, suggesting that certain specialties may inherently carry different levels of risk. This insight emphasizes the need for specialized risk assessments tailored to specific surgical contexts.
6. Height: Although less immediately intuitive, height was identified as a relevant predictor. It may serve as a proxy for other health metrics, potentially influencing surgical outcomes. This association suggests that further exploration into height-related health indicators may enhance risk assessment models.
7. Emergency Status (emergency): Emergency surgeries consistently demonstrated a higher risk of complications, likely due to limited preparation time and more severe underlying conditions in such cases. These findings suggest prioritizing risk mitigation strategies in emergency surgical protocols.
8. Diabetes Status (diabetes): Known to adversely affect wound healing and infection susceptibility, diabetes status was a significant factor in predicting complications. This underlines the importance of preoperative and postoperative management for diabetic patients to mitigate associated risks.

**Figure 7:**
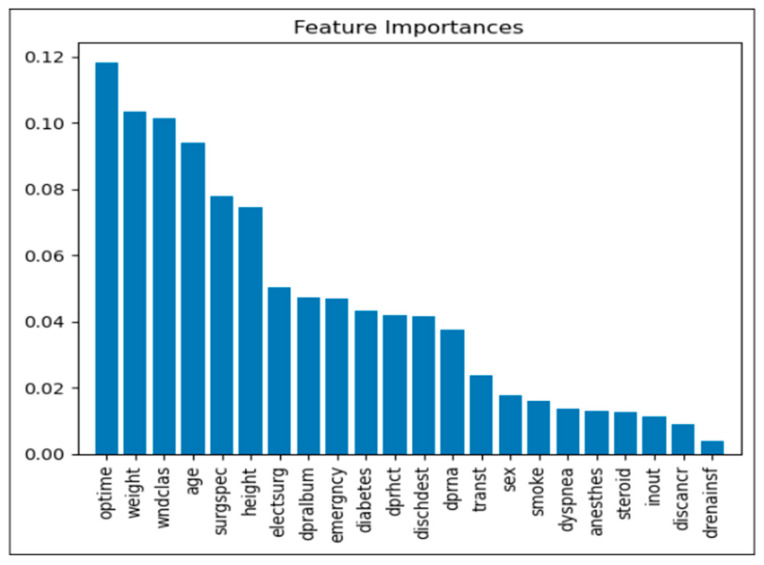
Feature Importance: Relative importance of key features in predicting postoperative complications. Operation time, weight, and wound classification were the most influential variables.

These findings provide a foundation for clinicians to better identify high-risk patients and implement tailored interventions. A clear understanding of feature importance supports the refinement of predictive models and the enhancement of patient care strategies. As shown in Figure 7, operation time, patient weight, and wound classification were the most influential predictors of postoperative complications, exhibiting the highest relative feature importance scores.

### 6.2. Demographic Influence

Demographic variables such as age and sex were also evaluated for their impact on the model’s predictions. Age was a substantial predictor, with older patients exhibiting a higher risk of postoperative complications. This observation aligns with the general pattern of increased health challenges among older adults. In contrast, sex demonstrated a moderate influence, with minor variations in prediction accuracy between male and female patients. These demographic insights may inform more precise, patient-centered approaches in risk assessment and clinical decision-making.

## 7. Interface Implementation and Usage Guide

The DiabCompSepsAI application has been developed to predict postoperative complications in diabetic patients, using a machine learning model within an intuitive, user-friendly interface.

### 7.1. Interface Implementation

#### Technology Stack

The DiabCompSepsAI interface is constructed using Streamlit, a platform that supports rapid web application development for machine learning models. This framework allows for straightforward interaction and deployment. The application employs key Python libraries: Pandas (Version 1.5.3) and NumPy (Version 1.24.3) for data manipulation and Joblib (Version 1.2.0) for loading the pre-trained model.

### 7.2. Model Integration

#### 7.2.1. Loading the Model

The predictive model, saved in Joblib format, is downloaded and seamlessly integrated into the application, enabling real-time predictions based on user input.

#### 7.2.2. User Input Handling

Categorical Inputs: For categorical variables, such as sex, inpatient/outpatient status, and surgical specialty, drop-down menus are implemented, allowing users to select options that are then mapped to numerical values through predefined dictionaries.

Numerical Inputs: Continuous variables, including age, height, weight, and operation time, are captured through direct input fields, ensuring accurate data entry.

#### 7.2.3. Prediction Process

Upon form submission, the application processes the inputs, converts them to the appropriate numerical format, and feeds them into the model. The model generates predictions indicating the likelihood of postoperative complications, specifically sepsis and wound infection. These predictions are then translated into clear, user-friendly messages for the end user.

### 7.3. User Instructions

#### 7.3.1. Getting Started

Access the Interface: Users can access the DiabCompSepsAI interface by visiting the provided application link. *Application Link*: https://diabcompsepsai.streamlit.app/.

Enter Patient Data: In the form, users are prompted to enter patient data by selecting from categorical options (e.g., sex, inpatient/outpatient status) and entering numerical values (e.g., age, height, weight, and operation time).

#### 7.3.2. Submitting Data

Once all fields are complete, users can submit the form by clicking the ‘Predict’ button.

The interface promptly displays the prediction results, indicating the presence or absence of postoperative complications. This real-time feedback allows healthcare providers to make quick, data-driven decisions regarding patient care.

The integration of the machine learning model with the Streamlit interface facilitates smooth interactions and delivers real-time predictions. This approach not only broadens access to the model but also enables healthcare professionals to evaluate patient risks more effectively, supporting improved clinical outcomes.

#### 7.3.3. Model Selection Rationale

In developing a predictive model for postoperative complications in diabetic patients, we selected the Random Forest Classifier due to several key advantages:

First, the Random Forest model demonstrated high accuracy and precision in predicting wound infection and sepsis, as verified by confusion matrices. It effectively reduces false positives while maintaining a high true negative rate. This level of reliability is critical in clinical settings.

Second, the Random Forest algorithms are well-suited for high-dimensional datasets, managing complex interactions between variables—an essential feature for analyzing the diverse variables in our dataset. This ensures that important relationships are not overlooked in prediction.

Third, the model provides insights into the relative importance of features, highlighting critical predictors such as operation time and weight, which are integral to understanding patient risk factors. It highlights predictors that align with clinical knowledge as well as those that may go unnoticed, enhancing clinical understanding.

Fourth, by leveraging ensemble learning and averaging across multiple decision trees, the Random Forest model minimizes overfitting, ensuring reliable performance on new data. This consistency and generalizability are invaluable when applying the model to diverse healthcare settings.

Fifth, the model balances interpretability and complexity, effectively managing both categorical and numerical data. This better facilitates its integration into clinical workflows, making it ideal for our application.

**Figure 8:**
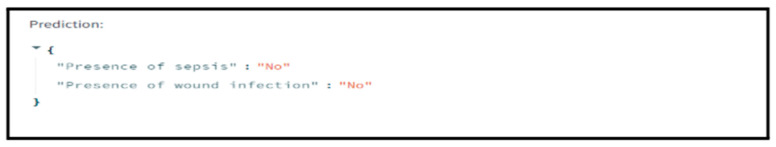
Prediction Preview: Screenshot of the DiabCompSepsAI application interface showing predicted outcomes and demonstrating the real-time predictive capabilities.

The choice of the Random Forest Classifier was driven by its robust performance, interpretability, and adaptability. These crucial factors enhance the model’s clinical relevance in assessing postoperative risks among diabetic patients. As shown in Figure 8, the DiabCompSepsAI interface presents a real-time preview of predicted outcomes, illustrating the application’s live predictive capabilities.

## 8. Discussion

In this study, we developed a novel machine learning application, DiabCompSepsAI, to predict postoperative wound infections and sepsis in diabetic patients. When trained and tested with a comprehensive dataset from the NSQIP, our Random Forest model demonstrated exceptional performance in terms of accuracy, precision, recall, and F1 score. This study presents promising evidence for this application to effectively identify high-risk patients in real-time and provide valuable insights regarding key features influencing postoperative complications.

Treating postoperative wound infection and sepsis in diabetic surgical patients is a critical challenge in perioperative care. Diabetes is known to impair wound healing and increase susceptibility to infections [11]. This means that accurate predictions of postoperative complications in diabetic patient populations are imperative, especially given the risks of increased morbidity, prolonged hospitalizations, and higher healthcare costs [12,13]. Existing prediction methods are limited by their accuracy, the number of variables considered, and their inefficient integration with clinical workflows [14,15,16].

In this study, we demonstrate the potential of machine learning techniques to address these limitations through a more user-friendly and reliable approach to risk prediction. Recent advancements in machine learning techniques have expanded the potential for improving predictions alongside the use of larger datasets with a wide range of variables. For example, studies using machine learning methods such as XGBoost have shown superior performance in predicting postoperative delirium compared to traditional regression models [17].

We selected the Random Forest Classifier for this context because of its performance and ability to handle complex datasets. The latter is especially important given the diverse range of predictors used in this study. Model development began with bootstrap sampling to create diverse training subsets of data. Random feature selection at each node reduces the correlation between trees and enhances the model’s generalizability. The model resists overfitting via ensemble learning and averaging over multiple decision trees.

Our research used the extensive NSQIP dataset, including over 1.5 million patient records across diverse surgical and perioperative scenarios, to train and validate the Random Forest model. After optimizing the model to predict wound infection and sepsis in diabetic patients, we found exceptionally high AUC-ROC data for wound infection and sepsis. What makes this model unique is its capacity to consider a wide range of relevant variables. These results highlight the growing potential of machine learning to capture insightful interactions between predictors and enable more personalized and effective perioperative management strategies.

This study furthers the field of machine learning predictive tools through our application. The seamless integration of the model into a user-friendly interface via Streamlit makes this tool widely accessible and allows real-time predictions. By allowing healthcare professionals to input patient data and instantly obtain a prediction, this application may serve as a valuable complement to current preoperative and postoperative management strategies, helping to improve patient outcomes and optimize resource allocation.

The DiabCompSepsAI application exhibits impressive performance, achieving accuracy rates of 94.08% for wound infection and 94.79% for sepsis. Similar high scores in precision and recall demonstrate that, in addition to correctly classifying true positives, this model minimizes false positives and false negatives, respectively. High precision helps avoid unnecessary treatment for patients who are not at risk. High recall ensures that patients requiring intervention are identified promptly, reducing the likelihood of complications progressing undetected. The model’s exceptional precision and recall scores are bolstered by F1 scores of 0.9342 for wound infection and 0.9432 for sepsis. These scores underscore a capability to balance precision and recall, which is crucial in ensuring that patients who are truly at risk are identified accurately and precisely.

The ROC-AUC scores for both wound infection and sepsis were notably high, further validating the model’s discriminative power. With an AUC of 0.92 for wound infection and 0.95 for sepsis, this model can effectively predict patients who will and will not have postoperative complications. This level of accuracy can significantly improve the early detection of postoperative complications, allowing for timely interventions and improved patient outcomes.

The feature importance analysis provided valuable insights into the factors most strongly associated with postoperative complications. The model’s identification of certain critical predictors aligns with established clinical understanding [18,19] and reinforces the model’s validity.

Operation time is a well-documented risk factor for postoperative complications [20,21,22] and was the most important predictor in the model’s analysis. This may not be a causal relationship, and there are myriad factors that may mediate the relationship between operation time and postoperative outcomes. For instance, extended operation times are associated with increased exposure to potential pathogens and physiological stress [23,24], both of which elevate the risk of complications. Risk is multifaceted, and exploring the links between predictors allows us to find applicable solutions. Surgical specialty, the fifth most important predictor of postoperative complications, is another potential perioperative confounder. It is essential to tailor risk assessments by specialty, as these results suggest that the surgical context affects risk [25].

Patient weight was the second most influential predictor. The association of numerous health conditions with obesity [26] may mediate this correlation, stressing the importance of weight management for patients. Wound classification was the next most important variable. It was no surprise that contaminated wounds correlated with an increased risk for infection and sepsis. This relationship emphasizes the importance of wound management. The analysis highlighted that age was the fourth most important feature, with older patients at an elevated risk of complications likely due to the increased prevalence of comorbidities in elderly populations [27]. Surgeries in an emergency context were a significant predictor of post-operative complications. This may be due to the urgent nature of the operations, which suggests a review of risk management strategies in emergency surgery settings [28]. The model also indicated diabetic status as an influential predictor. As discussed, the literature affirms that diabetes decreases rates of wound healing and increases susceptibility to infection, which necessitates better planning around post-operative care for diabetic patients.

Interestingly, our model also uncovered a few unexpected variables as significant predictors that may otherwise be overlooked. Height was revealed as a significant but less intuitive predictor. This finding warrants further investigation into the role of patient height and related measures that may be associated with postoperative outcomes.

One of the key strengths of the DiabCompSepsAI application lies in its ability to integrate complex data and derive actionable insights. This ability underscores the value of machine learning in identifying patterns that conventional methods are prone to miss [29]. These insights will better inform future targeted interventions [30].

### 8.1. Limitations

While the DiabCompSepsAI application demonstrates strong performance, it is important to acknowledge its limitations. The model was developed and validated using a retrospective dataset from the NSQIP, which may not fully capture the complexities of real-world clinical practice. External validation of the model in clinical settings is necessary to assess its generalizability.

Although the NSQIP dataset provides a solid foundation for model development, certain clinically relevant variables, such as specific intraoperative events or detailed postoperative care protocols, are not accounted for. The model is reliant on a set of features that may not fully capture the nuances of individual patients. Incorporating additional predictors may potentially improve the model’s predictive accuracy [31]. Additionally, the model was trained and validated on a dataset of patients from the U.S. Our model then faces issues with generalizability in other regions where differences in healthcare settings and patient demographics could influence the model’s performance.

Our model assumes that variables are consistently recorded and readily available in clinical practice. In reality, there can be great variability in data quality and completeness across institutions [32], which may pose challenges for implementation in the real world.

Another limitation lies in the complexity of machine learning models, which can hinder their interpretability and acceptance among clinicians. This ‘black box’ nature may pose challenges for integration into routine practice. Predictive model transparency would allow clinicians to better understand the model and its underlying drivers of risk prediction. This interpretability facilitates trust and adoption among healthcare personnel [33].

### 8.2. Future Directions

The findings suggest actionable steps to improve patient care, such as optimizing surgical efficiency and enhancing perioperative management for diabetic patients. Prospective studies should focus on external validation across diverse patient populations and healthcare systems. Datasets from various settings and demographics will ensure robustness and generalizability, addressing potential biases.

Future research should also leverage machine learning to deepen our understanding of the pathophysiological mechanisms behind postoperative complications in diabetic patients. Identifying patterns in the data may lead to new preventive or therapeutic strategies to reduce the burden of wound infections and sepsis.

Improving model interpretability is essential for clinician adoption. Techniques like individual condition expectation plots could explain how feature changes impact predictions [15,34]. This would facilitate understanding and enhance adoption in clinical practice.

Pilot studies and clinical trials are necessary to evaluate the model’s impact on patient outcomes and clinician decision-making, measuring both quantitative and qualitative factors such as user satisfaction. Once validated, integrating the model into EHR systems could streamline implementation and facilitate real-time predictions, reducing manual data entry, as demonstrated in other perioperative machine learning applications [35,36].

Addressing these directions will enhance the DiabCompSepsAI model’s potential to improve clinical outcomes and advance our understanding of postoperative complications in diabetic patients, ultimately improving care quality.

## 9. Conclusions

Our study represents a significant advancement in the prediction of postoperative complications in diabetic patients. By leveraging the power of machine learning, the DiabCompSepsAI application offers a valuable tool for clinicians to identify high-risk patients and implement targeted interventions. While further research is needed for validation, the potential to improve patient outcomes and reduce healthcare costs is significant. As machine learning continues to evolve, we anticipate further advancements in the development of predictive models that will revolutionize postoperative care.

## Figures and Tables

**Table 1:** Performance Metrics for Predictive Model on Wound Infection (wndinf) and Sepsis (prsepis).

Metric	Wndinf	Prsepis
Accuracy	0.9408	0.9479
Precision	0.9361	0.9430
Recall	0.9408	0.9479
F1 Score	0.9342	0.9432

## Data Availability

The data that was used for the findings of this study are derived from the American College of Surgeons National Surgical Quality Improvement Program (ACS NSQIP). Due to data use agreements and privacy regulations, these data are not publicly available. Researchers interested in accessing NSQIP data must obtain permission and licensing directly from the American College of Surgeons through their NSQIP Participant Use File program. https://www.facs.org/quality-programs/data-and-registries/acs-nsqip (accessed on 23 August 2023).
